# Case Report: Nebulized autologous platelet-rich plasma associated with quantitative CT and clinical changes in a patient with COPD and chronic hypersensitivity pneumonitis

**DOI:** 10.3389/fmed.2026.1939351

**Published:** 2026-09-22

**Authors:** Zane Sherif, Amanda Forbes, Kirralee Sherif

**Affiliations:** Mermaid Beach Radiology, Gold Coast, QLD, Australia

**Keywords:** case report, chronic obstructive pulmonary disease (COPD), emphysema, hypersensitivity pneumonitis (HP), nebulization, platelet-rich plasma (PRP), quantitative computed tomography (QCT)

## Abstract

**Background:**

Chronic obstructive pulmonary disease (COPD) and chronic hypersensitivity pneumonitis are characterised by progressive structural lung damage, declining respiratory function, and limited regenerative treatment options. Platelet-rich plasma (PRP) possesses anti-inflammatory and regenerative properties; however, its pulmonary application remains largely unexplored.

**Case presentation:**

A 70-year-old man with a longstanding history of COPD and chronic hypersensitivity pneumonitis underwent eight treatments with nebulized autologous PRP at approximately 4-week intervals. In this observational case report, changes during the treatment period were assessed using serial clinical CT imaging with quantitative analysis, pulmonary function testing, home peak expiratory flow (PEF) and patient-reported outcomes.

**Results:**

At follow up, qCT demonstrated a decrease in low attenuation area percentage (LAA%) from 17.4% to 13.6%, representing a 22% relative reduction, accompanied by an 11.5% reduction in total inspiratory lung volume. Formal spirometry PEF increased by approximately 20% compared to the most recent pre-treatment assessment, with similar increases observed on home PEF monitoring. The patient also reported marked reductions in exertional dyspnea, substantially improved walking tolerance, and increased energy levels. Nebulized PRP was well tolerated, with no adverse events attributed to treatment.

**Conclusion:**

To our knowledge, this is the first report describing serial qCT assessment alongside physiological and patient-reported outcomes following nebulized autologous PRP in an individual with COPD and chronic hypersensitivity pneumonitis. Although causality cannot be established from a single case, the findings across multiple outcome domains warrant further investigation in prospectively designed studies.

## Introduction

Chronic obstructive pulmonary disease (COPD) and chronic hypersensitivity pneumonitis are respiratory disorders associated with progressive structural lung injury, impaired exercise tolerance, reduced quality of life, and increased mortality. Current treatment strategies focus primarily on symptom management, prevention of disease progression, and avoidance of ongoing environmental exposures. Established structural lung damage, including emphysematous and fibrotic change, is generally considered irreversible (1, 2).

Platelet-rich plasma (PRP) is a blood-derived biologic containing concentrated platelets and numerous bioactive growth factors involved in tissue repair, angiogenesis, and modulation of inflammation. While PRP has been widely used in regenerative medicine across numerous clinical disciplines, its application in pulmonary disease remains relatively unexplored. Emerging preclinical and clinical evidence suggests potential benefits in lung injury and chronic respiratory disease, including improvements in pulmonary function, inflammatory responses, and radiographic findings. These observations have been reported in preclinical studies, early clinical investigations, and recent case reports of nebulized PRP therapy (3, 4).

We present an observational case report of a patient with COPD and hypersensitivity pneumonitis (Bird Fancier’s Lung) who underwent eight nebulized autologous PRP treatments at approximately 4-week intervals, with longitudinal assessment using quantitative computed tomography (qCT), serial home peak expiratory flow measurements, formal spirometry and patient-reported functional outcomes.

## Case presentation

This observational case report describes the clinical course of a 70-year-old man with longstanding history of chronic obstructive pulmonary disease (COPD) and chronic hypersensitivity pneumonitis (Bird Fancier's Lung), reportedly diagnosed approximately 15 years previously, without a documented history of pulmonary fibrosis. He presented to our clinic for consideration of investigational nebulized autologous PRP as an adjunct to his existing clinical management. These pre-existing diagnoses were documented in correspondence from his usual treating respiratory specialist; however, detailed records of original diagnostic work-up for his lung conditions were not available to the authors.

Relevant comorbidities reported by the patient included chronic sinusitis, gastro-oesophageal reflux disease, and gout. The patient reported a previous bird-keeping hobby but had ceased avian exposure approximately three years prior to presentation, with no further exposure reported during the observation period. He had a limited smoking history of 0.5 pack-years, having ceased smoking at age 22. He also reported previous occupational dust exposure, which had ceased at retirement, with no further dust exposure during the observation period.

The patient was under regular respiratory specialist review. Correspondence from his usual treating respiratory specialist documented stable diffusing capacity for carbon monoxide (DLCO) of approximately 48% predicted. The specialist considered the patient's respiratory condition unlikely to improve, but also unlikely to progress in the absence of further avian antigen exposure.

The same correspondence documented significant exertional dyspnea and limited functional capacity requiring rest after walking approximately 20 metres on level ground. Chronic productive morning cough and sputum production were also documented and were considered predominantly related to co-existing chronic sinusitis and were managed through short course of antibiotics. At presentation to our clinic, the patient also reported persistent fatigue and low energy levels as well as alcohol consumption of up to 20 standard drinks per week.

The patient had prior documentation of emphysematous changes and pulmonary nodules which had been monitored qualitatively at approximately 6–12 month intervals external to our clinic, as a part of his ongoing clinical care. Based on available prior specialist correspondence and radiology reports, there was no documented diagnosis of pulmonary fibrosis.

At baseline, the patient's respiratory condition was managed with a combination inhaler of fluticasone furoate/umeclidinium/ vilanterol (Trelegy Ellipta) twice daily and prophylactic clarithromycin. Baseline concomitant medications and supplements are listed in Table 1.

**Table 1:** Concomitant medications.

Medication/supplement	Baseline regimen	Indication	Change during observation period
Fluticasone furoate/umeclidinium/vilanterol (Trelegy Ellipta)	BD	Maintenance inhaled respiratory therapy	Patient independently reduced to one inhalation once daily.
Clarithromycin (250 mg)	BD	Prophylactic therapy	No change
Aspirin (100 mg)	Daily	Antiplatelet therapy	Discontinued∼2 weeks before treatment initiation
Irbesartan/hydrochlorothiazide (Avapro HCT, 300/12.5 mg)	Daily	Antihypertensive therapy	No change
Diltiazem hydrochloride (Cardizem CD, 250 mg)	Daily	Cardiovascular therapy	No change
Simvastatin (40 mg)	Daily	Lipid-lowering therapy	No change
Paroxetine (20 mg)	Daily	SSRI	No change
Esomeprazole (Nexium, 20 mg)	Daily	Proton-pump inhibitor	No change
Allopurinol (Zyloprim, 300 mg)	Daily	Gout management	No change
Vitamin B	Daily	Supplement; formulation unknown	No change
Vitamin D	Daily	Supplement; formulation unknown	No change
Krill oil (500 mg)	Daily	Supplement	No change
Milk thistle (500 mg)	Daily	Supplement	No change

### Treatment

Immediately prior to each treatment, autologous PRP was prepared under aseptic conditions from 120 mL of peripheral whole blood using a commercially available validated centrifugation system (EmCyte Corporation, USA), in accordance with the manufacturer's 120-mL preparation protocol without exogenous activation. The final volume of PRP was approximately 20 mL; however, baseline whole-blood and final PRP platelet and leukocyte counts were not measured in this case. Based on the standardized preparation protocol, the expected formulation corresponds with PAW classification P4-Aα (5). The leukocyte-rich PRP formulation reflected our established clinical practice and prior publications (4) and was not specifically selected on the basis of proposed pulmonary advantage over leukocyte-poor PRP.

Autologous PRP was nebulized using a compressor-driven jet nebulizer system (InnoSpire Deluxe, Philips Healthcare, Netherlands). The device is approved by Therapeutic Goods Administration (TGA) for aerosol delivery of medications for respiratory therapy (ARTG 278359); however, its use for autologous blood products is outside the manufacturer's stated intended use. Prior to each treatment, the patient nebulized an ampule of salbutamol (1 mg/mL). Approximately 20 mL of PRP was administered by face mask at each treatment over approximately 60 min. The patient remained seated upright during administration, breathing normally. A small amount of residual PRP was visibly deposited within the treatment mask and delivery tubing following administration; however, was not quantified.

During and immediately following administration, the patient remained under direct nursing observation. Treatment tolerability was assessed through clinical observation and patient enquiry regarding new or worsening respiratory or systemic symptoms, including cough, shortness of breath or wheeze. Inter-treatment adverse events were assessed at subsequent treatment visits through patient enquiry regarding any new or worsening symptoms since the last treatment.

The patient returned to our clinic for treatment every four weeks (± 1 week) for a total of eight sessions. One scheduled treatment was deferred due to confirmed human rhinovirus (HRV) respiratory illness. Treatment resumed once the patient had clinically recovered and returned to baseline health status. The treatment course and timing of clinical assessments are summarized in Figure 1.

**Figure 1:**
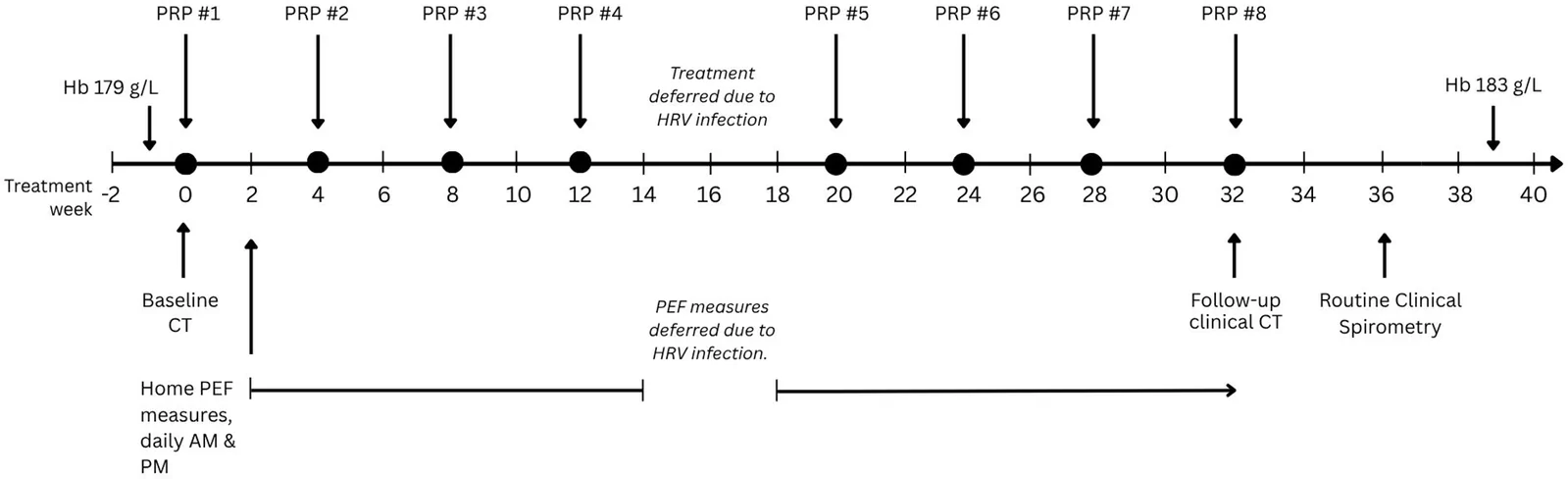
Timeline of nebulized autologous platelet-rich plasma (PRP) treatment and assessments. The patient underwent eight treatments at approximately 4-week intervals. The timing of CT imaging, peak expiratory flow (PEF) monitoring, routine pulmonary function assessments, and available hemoglobin measurements is shown relative to treatment initiation at week 0. One scheduled treatment was deferred due to human rhinovirus (HRV) respiratory infection.

Aspirin therapy was discontinued two weeks prior to treatment initiation to minimise potential effects on platelet function and PRP preparation. The patient was additionally advised to avoid non-steroidal anti-inflammatory drugs (NSAIDs) and limit alcohol consumption throughout the treatment period. As treatment progressed, the patient independently reduced his inhaled respiratory medication therapy from two inhalations twice daily to one inhalation once daily. No other changes were made to his prescribed medications during the observation period (Table 1).

This case report was prepared in accordance with the CARE guidelines.

### Assessments

Outcomes assessed during the observation period included quantitative computed tomography (qCT) imaging, serial home peak expiratory flow (PEF) measurements, pulmonary function tests, and patient-reported respiratory symptoms and functional capacity.

Computed tomography (CT) imaging was performed in accordance with our institutional practice for routine chest CT, using the same IQon Spectral CT (Philips Healthcare, Netherlands) and acquisition and reconstruction protocols. Examinations consisted of a single-phase, unenhanced high-resolution acquisition at full inspiration during a single breath hold with images reconstructed using monoE40 and electron-density spectral maps. No expiratory acquisitions were performed, consistent with our routine institutional protocol.

Baseline and follow-up CT imaging were performed on the respective treatment days, immediately prior to nebulized PRP administration. Both examinations were acquired for clinical purposes, with follow-up imaging requested by the patient's respiratory specialist as part of ongoing clinical care. Follow-up imaging coincided with the eighth treatment visit, approximately 32 weeks after baseline.

For qCT measures, automated segmentation and volume quantification of lung tissue were performed using Advanced Visualisation Workspace (AVW) CT COPD post-processing software (Philips Healthcare, Netherlands). Emphysematous change was quantified using a low attenuation area (LAA) threshold of −950 Hounsfield Units (HU). The volume of lung parenchyma below −950 HU was calculated in cubic centimetres and expressed as a percentage of total lung volume (LAA%). Functional small airway disease (fSAD%) was not calculated as paired inspiratory and expiratory CT datasets were not available.

Home peak expiratory flow (PEF) was monitored from weeks 2 to 36 using a Breath-Alert^TM^ peak flow meter (Allersearch Laboratories, USA) twice daily through the observation period. The mean of three readings was recorded in the morning and evening at approximately the same times each day. An approximately three-week interruption in home PEF measures occurred during a viral respiratory illness (human rhinovirus).

As part of the patient's routine respiratory care and specialist review, the patient underwent routine spirometry at an accredited respiratory laboratory independent of the treating clinic. This assessment coincided with approximately week 36 of the observation period and was compared with the most recent available pre-treatment spirometry from May 2025 (approximately 5 months earlier), performed at the same laboratory.

Functional capacity was evaluated descriptively through patient-reported changes in walking tolerance during the observation period. As this was an observational case report of clinical care, no standardized exercise testing, such as a six-minute walk test, was introduced specifically for research purposes.

### Outcomes

Baseline high resolution CT (HRCT) demonstrated emphysematous hyperlucency and bullous changes which were described as severe by the reading radiologist (Figure 2). Follow-up imaging demonstrated persistent extensive emphysematous changes with a subtle increase in interstitial attenuation. There was no CT evidence of pulmonary fibrosis at either timepoint. Representative baseline and follow-up qCT images with automated lobar segmentation and low-attenuation areas are shown in Figure 3.

**Figure 2:**
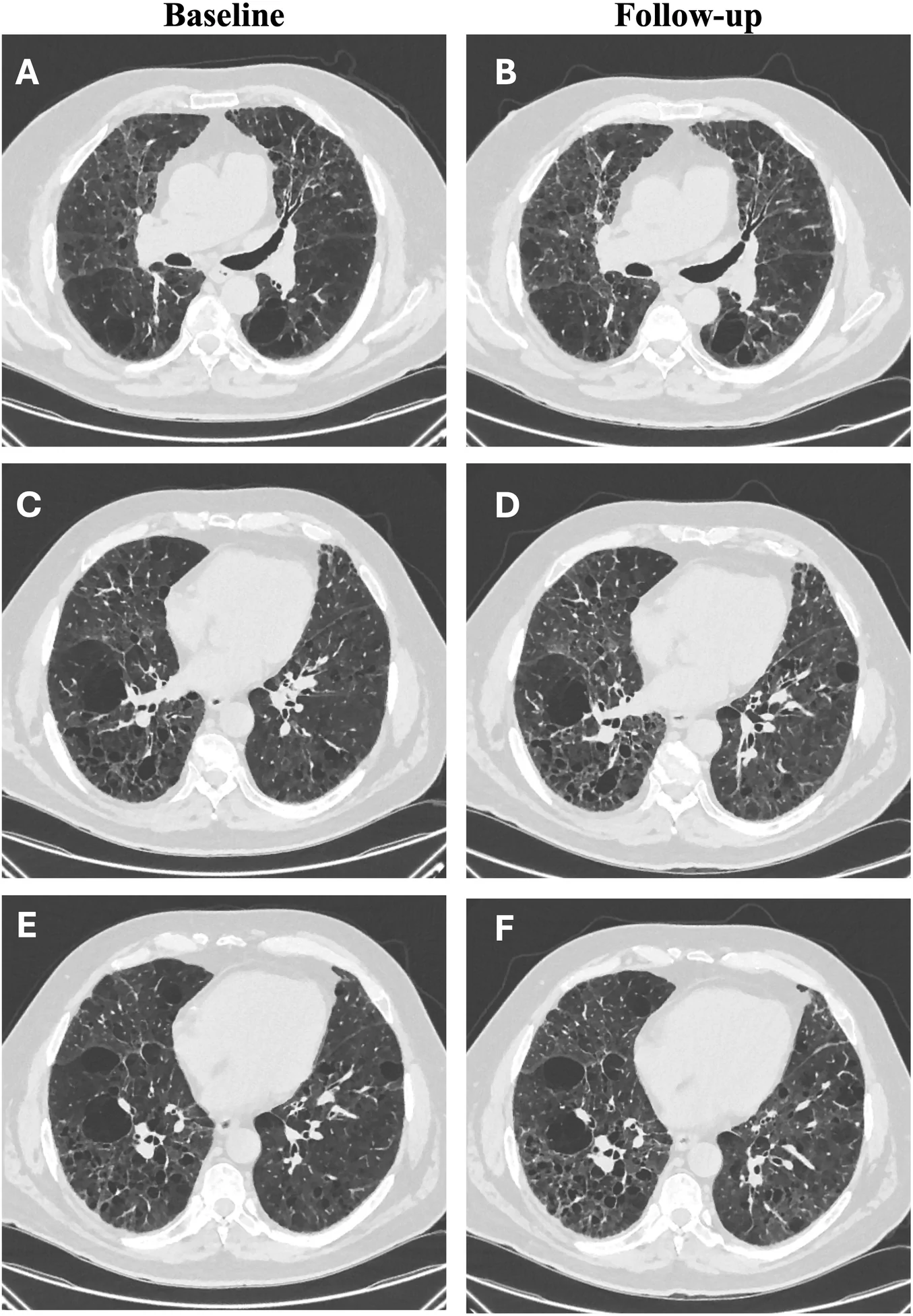
Representative high-resolution CT (HRCT) images at baseline **(A,C,E)** and at follow-up **(B,D,F)**. Axial inspiratory images, acquired as part of routine clinical care, demonstrate persistent extensive emphysematous and bullous changes, with no CT evidence of pulmonary fibrosis at either timepoint. Subtle visual differences in low attenuation and an increase in denser parenchymal markings were noted at follow-up. Images are displayed with standard window settings (W:1600, L: −600 HU).

**Figure 3:**
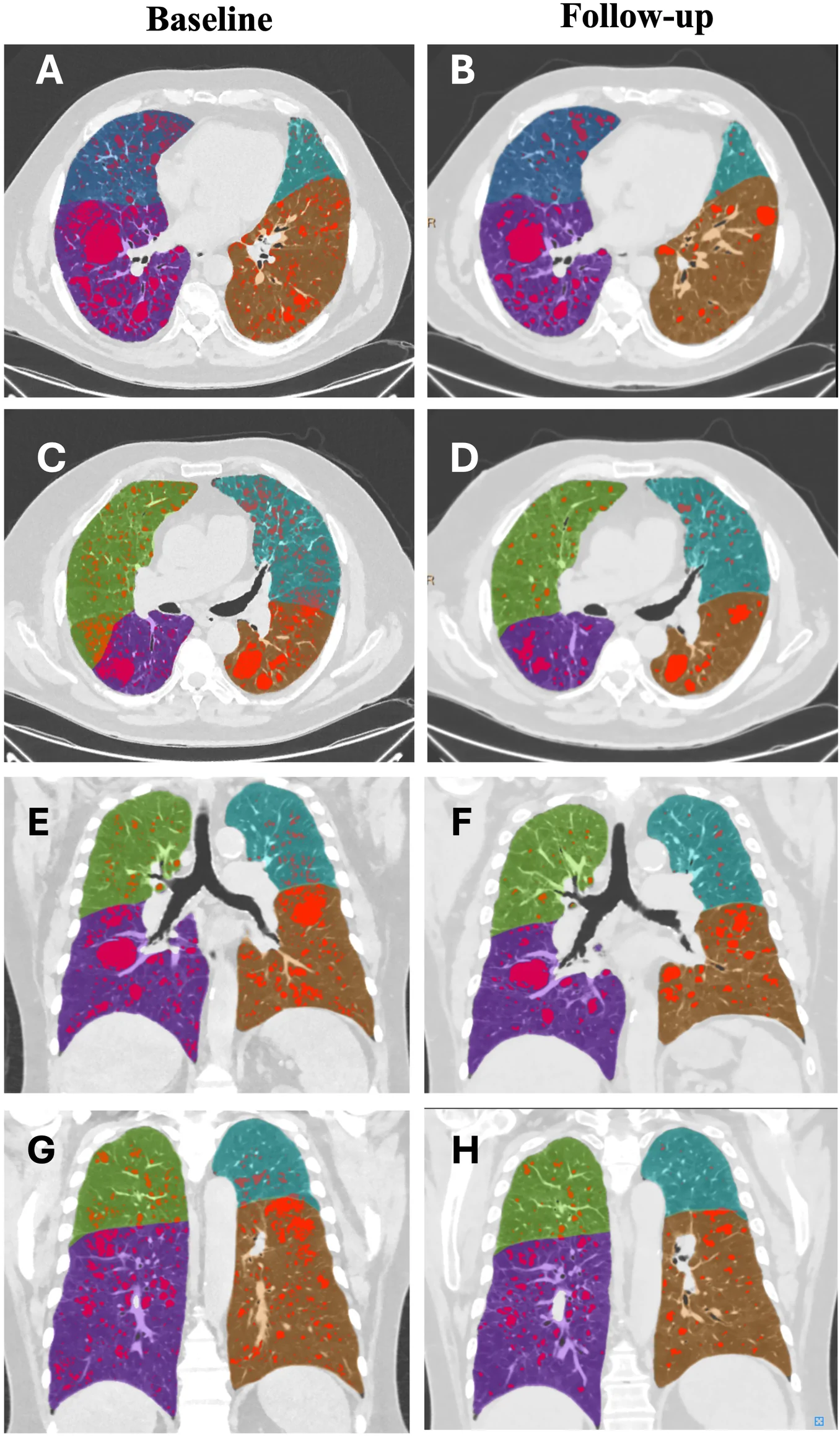
Quantitative computed tomography (qCT) assessment at baseline **(A, C, E, G)** and at follow-up **(B, D, F, H)**. Representative axial (**A–D**) and coronal (**E–H**) inspiratory qCT images demonstrate automated lobar segmentation, with red regions indicating low-attenuation areas (< −950 HU), corresponding to areas of hyperlucency and bullous changes. Expiratory acquisitions were not obtained.

Compared with baseline imaging, qCT analysis demonstrated a reduction in total lung volume (11.5%) and a decrease in low attenuation area percentage (LAA%) from 17.4% at baseline to 13.6% on follow-up imaging, representing a relative reduction of approximately 22%. Regional analysis demonstrated that the greatest reduction in LAA% occurred within the left lower lobe (27%) (Table 2).

**Table 2:** Inspiratory quantitative CT analysis at baseline and follow-up.

Region	LAA volume (cc)			Total volume (cc)			LAA %		
	Baseline	Follow-up	Change (%)	Baseline	Follow-up	Change (%)	Baseline	Follow-up	Change (%)
Both Lungs	1158.5	803.2	−30.7	6652.0	5885.2	−11.5	17.4	13.6	−21.8
R Lung	597.4	428.0	−28.4	3468.3	3129.0	−9.8	17.2	13.7	−20.3
RUL	141.4	110.5	−21.9	1167.5	1055.8	−9.6	12.1	10.5	−13.2
RML	81.3	65.2	−19.8	484.8	465.1	−4.1	16.8	14.0	−16.7
RLL	374.7	252.3	−32.7	1816.0	1608.1	−11.4	20.6	15.7	−23.8
L Lung	561.1	375.2	−33.1	3183.8	2756.2	−13.4	17.6	13.6	−22.7
LUL	235.8	170.3	−27.8	1389.3	1206.2	−13.2	17.0	14.1	−17.1
LLL	325.3	205.0	−37.0	1794.5	1550.0	−13.6	18.1	13.2	−27.1

Serial home peak expiratory flow (PEF) measurements increased over the observation period. Comparison of the early and late treatment periods (weeks 2–5 and 33–36, respectively) demonstrated a 19.5% increase in mean morning PEF (418–499.5 L/min). A similar increase was observed in evening PEF (19.2%) (Figure 4).

**Figure 4:**
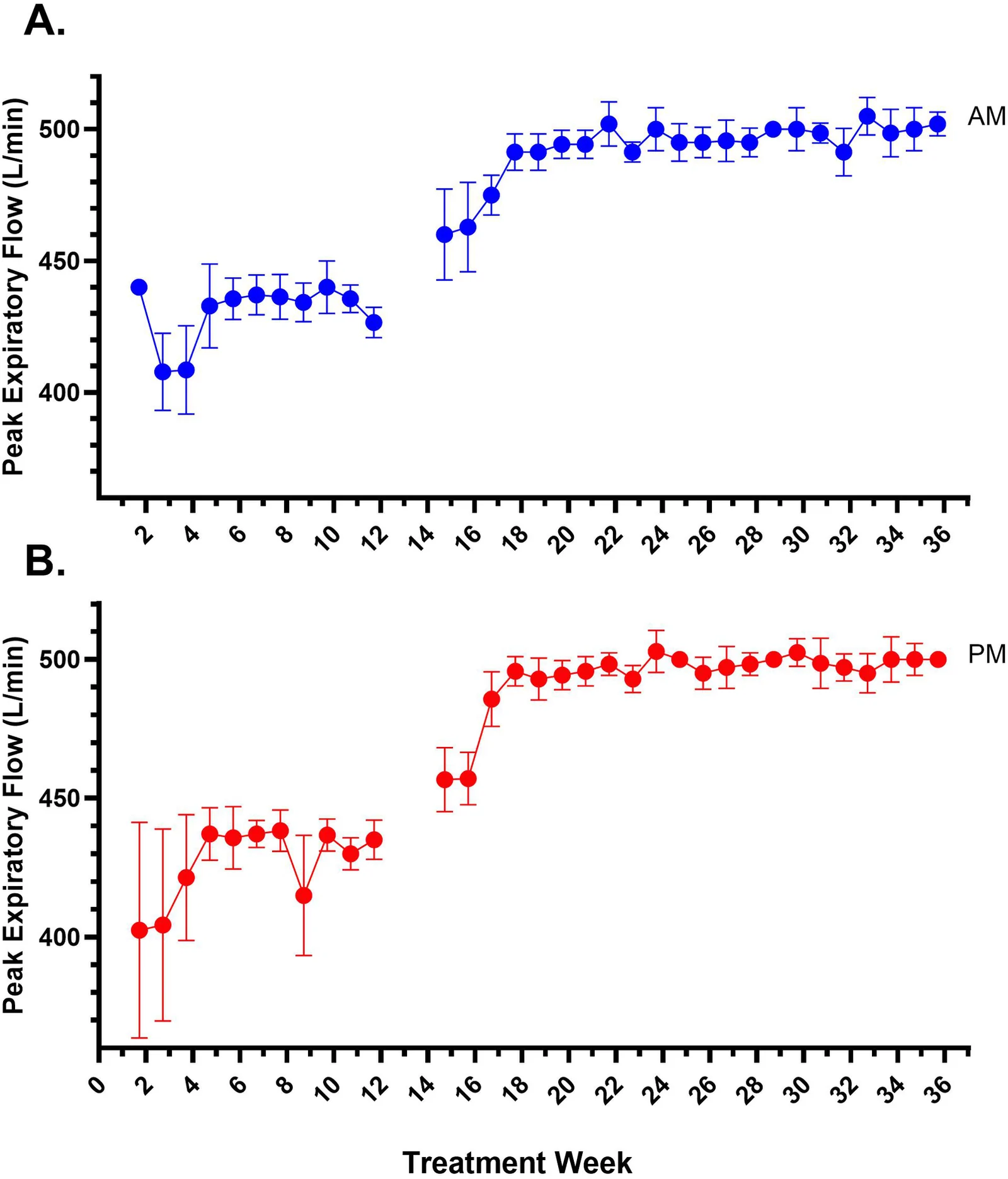
Weekly mean home peak expiratory flow (PEF) measurements recorded in the morning **(A)** and evening **(B)** during weeks 2 to 36 of the observation period. Data are presented as weekly mean ± standard deviation (SD). An approximately three-week interruption in data collection occurred during recovery from human rhinovirus (HRV) respiratory illness.

Formal spirometry was performed independently as a part of the patient's routine respiratory review which coincided with approximately week 36 of the observation period. Results were compared with the most recent available pre-treatment spirometry (Table 3). FEV_1_ and FVC remained relatively stable, while PEF increased by approximately 20%, consistent with the pattern observed in serial home PEF measures. In contrast, measured alveolar volume (VA) and single-breath diffusing capacity (DLCO) and calculated KCO decreased by 6.3%, 14.6%, and 8.8%, respectively (Table 3). Available hemoglobin measurements, obtained through an independent pathology as part of routine clinical care, were similar at approximately one week prior to commencement of treatment and at week 39 (179 and 183 g/L, respectively) (Figure 1). No contemporaneous hemoglobin measurement was available at the time of pre-treatment pulmonary function assessment.

**Table 3:** Comparison of pre-treatment and follow-up lung function assessments.

Measure	Pre-treatment			Follow-up			
	Post-BD *27 May 2025*	% of predicted	Z-score^a^	Post-BD *06 Jul 2026*	% of predicted	Z-score^a^	% change
FEV_1_ (L)	2.42	84.1	−0.94	2.41	84.7	−0.86	−0.4
FVC (L)	3.61	96.3	−0.33	3.64	97.7	−0.30	0.7
FEV_1_/ FVC (%)	67.06	89.9	−0.98	66.33	89.1	−0.89	−1.1
PEF (L/min)	561.66	121.6	1.00	674.34	146.8	3.42	20.1
DLCO single-breath ml/min/mmHg^b^	12.22	48.4	–	10.44	41.7	–	−14.6
VA single-breath (L)	6.30	96.7	–	5.9	90.6	–	−6.3
KCO^c^	1.94	–	–	1.77	–	–	−8.8

Pre-treatment assessment was the most recent available pulmonary function test and was performed approximately five months prior to initiation of nebulized PRP. Both assessments were performed at the same independent respiratory laboratory as a part of routine clinical care. Predicted values were reported using GLI2012 and GLI 2020 reference equations for DLCO. Spirometry reference values were adjusted for ethnicity; DLCO from Caucasian populations. Spirometry and DLCO manoeuvres were noted as acceptable/reproducible. Measures obtained post-bronchodilator (terbutaline sulfate) (Post-BD).

a Z-score not available for DLCO, VA or KCO.

b DLCO not corrected with hemoglobin.

c KCO values were calculated by the authors from reported DLCO and VA measures (KCO = DLCO/VA) and were not provided by laboratory.

During the observation period, the patient reported progressive improvement in exertional dyspnea and walking tolerance. Walking tolerance over-ground increased from approximately 20 m at baseline to approximately 460 m coinciding with week 32. At approximately week 39, the patient reported being able to walk up to 1,400 m on a treadmill without requiring rest. As these observations were obtained under different, non-standardized walking conditions, they are presented as descriptive observations and are not directly comparable. The patient also reported improvements in productive morning cough and fatigue, with an increase in motivation and energy levels compared to baseline. No adverse events attributed to nebulized PRP were reported by the patient during the observation period.

## Discussion

This case report describes a 70-year-old man with a longstanding history of COPD and hypersensitivity pneumonitis (Bird Fancier's Lung) who underwent eight treatments of nebulized autologous PRP at approximately 4-week intervals. During the treatment period, changes were observed across multiple assessment modalities, including objective quantitative CT (qCT) imaging, peak expiratory flow (PEF), as well as patient-reported respiratory symptoms and functional capacity.

While spirometry remains fundamental to the assessment of chronic lung disease, qCT provides complementary structural biomarkers that may better reflect the anatomical consequences of emphysema and hyperinflation (6, 7). LAA threshold of −950 HU (LAA_−950_) is a validated and highly reproducible CT biomarker of emphysema when assessed using standardized inspiration protocols (8). The observed qCT changes in the present case are therefore of particular interest, as they may indicate reductions in disease-associated hyperinflation and emphysematous burden—two key contributors to dyspnea and functional limitation in COPD (9). However, hyperinflation and gas trapping were not directly assessed, and LAA% (−950 HU) is known to be sensitive to differences in inspiratory lung volumes (8). In a controlled study of patients with COPD, the reduction in inspiratory level from 100% to 90% of vital capacity resulted in approximately 2% difference in the percentage of lung below −950 HU (10). As such, differences in lung inflation between CT scans may have contributed to the reduction in LAA%. However, the magnitude of their contribution to the observed change cannot be determined.

The observed increase in PEF on serial home monitoring was also reflected in formal spirometry, while FEV₁ and FVC remained essentially unchanged from the most recent pre-treatment assessment. Together with patient-reported improvements in respiratory symptoms and functional capacity, these findings raise the possibility of improved expiratory function during the treatment period. In COPD, emphysema reduces lung elastic recoil, diminishing expiratory driving pressure and promoting airflow limitation through narrowed, poorly supported airways (11). Accordingly, the observed improvement in PEF may reflect changes in airway mechanics associated with reduced airway inflammation, oedema, mucus burden, and hyperinflation. Such mechanisms are biologically plausible given the recognized anti-inflammatory and immunomodulatory properties of PRP (12) and are consistent with previous reports of reduced airway inflammation and mucus production following nebulized PRP administration in smoke inhalation injury (13).

Not all physiological measures changed in a favorable direction. At follow-up, DLCO and alveolar volume (VA) declined alongside a reduction in inspiratory CT lung volume. The reductions in VA and inspiratory lung volume may be consistent with reduced hyperinflation; however, the overall pattern of reduced lung volume, VA, and DLCO is non-specific and, in the context of chronic hypersensitivity pneumonitis, progression of underlying interstitial disease remains an alternative consideration. There was no CT evidence of pulmonary fibrosis at baseline or follow-up. The clinical significance of these physiological changes therefore remains uncertain.

Clinically, the patient also reported reduced exertional dyspnea, markedly improved walking tolerance, and increased energy levels. Although causal inference cannot be established from a single case, favorable changes across structural imaging, PEF, and patient-reported outcomes are encouraging, despite the discordant changes in gas-transfer measures. Such findings are noteworthy given that both COPD and chronic hypersensitivity pneumonitis are typically characterized by progressive functional limitation and structural lung changes that are generally considered largely irreversible (1, 2).

The findings of the present case can be considered in the context of an emerging body of literature describing pulmonary applications of PRP in both chronic and acute lung disease. However, clinical evidence remains preliminary and methodologically heterogeneous, with limited controlled data and studies predominantly evaluating intravenous (IV) PRP. Improvements in pulmonary function, quality of life, and clinical outcomes have been reported in COPD and COVID-19-associated acute respiratory distress syndrome without serious treatment-related adverse events (14–18); however, theoretical thrombotic concerns associated with systemic administration have also been raised (14–18).

Nebulized PRP represents an alternative route of administration that may reduce the theoretical thrombotic risks of systemic administration while directly targeting diseased pulmonary tissue. *In vitro* studies have demonstrated comparable biological activity between standard and aerosolized PRP, supporting preservation of key growth factors following nebulization (19). Preliminary clinical evidence has reported reduction in airway inflammation, oedema, mucus production, and shorter intubation and hospitalisation durations following nebulized autologous PRP in patients with smoke inhalation injury (13). More recently, the nebulized PRP *Sherif Pulmonary Protocol* was associated with radiological resolution of persistent pulmonary consolidation and post-infective cavitation following nebulized autologous PRP in a patient with prior thoracic irradiation (4). Collectively, these studies provide a rationale for further investigation but do not establish efficacy or a regenerative mechanism. Rigorously designed prospective controlled studies are required to determine the safety, efficacy and potential biological effects of nebulized PRP in pulmonary disease.

The optimal PRP formulation for pulmonary administration remains undefined. Although preclinical studies have suggested leukocyte-rich PRP may promote inflammatory signalling, this pro-inflammatory effect has not been consistently demonstrated in clinical studies (20). Importantly, the cellular and molecular composition of the generated aerosol, including platelet and leukocyte integrity, was not characterized in the present case; therefore, the extent to which intact cells and their associated mediators were aerosolized remains unknown. Additionally, the extent of pulmonary deposition and delivery of aerosolized PRP to the distal airways was not assessed. As such, further studies are required to characterize nebulized PRP, assess its pulmonary deposition and distribution, and determine the relative pulmonary effects of leukocyte-rich and leukocyte-poor formulations.

Several additional limitations of this observational case report warrant consideration. As an observational single-case report without a comparator, causality cannot be established. The patient independently reduced his inhaled corticosteroid-containing therapy during the observation period. While this reduction does not readily explain the observed favorable outcomes, its potential influence cannot be excluded and represents a confounding factor of uncertain significance. Symptom and functional outcomes were self-reported rather than assessed using standardized exercise testing or validated quality-of-life measures. As imaging was acquired as part of routine clinical care, CT assessments were limited to inspiratory acquisitions, precluding quantitative assessment of expiratory air trapping and functional small airway disease. Physiological findings were not uniformly favorable, with lower DLCO and VA at follow-up, the significance of which remains uncertain. Interpretation of the change in DLCO is further limited by the absence of hemoglobin-corrected measurements at the time of the pre-treatment pulmonary function assessment. As such, prospectively controlled studies are required to evaluate the safety, biological effects and clinical efficacy of nebulized PRP in chronic respiratory disease.

## Conclusion

To our knowledge, this is the first report describing serial qCT imaging alongside physiological and clinical changes following eight nebulized autologous PRP treatments at approximately 4-week intervals in a patient with COPD and chronic hypersensitivity pneumonitis. Although causal inference cannot be established from a single-patient observation, favorable changes in quantitative imaging, PEF, and patient-reported outcomes where observed. Nebulized PRP was well tolerated throughout the treatment period, with no treatment-related adverse events observed. These findings contribute to the limited literature investigating pulmonary applications of PRP and support further mechanistic and prospective clinical studies of nebulized PRP in chronic respiratory disease.

### Patient experience

I am elated by the effect of Nebulized PRP Treatment as I no longer have a chronic productive cough and breath much freer, I can walk much further than before the treatment and I generally feel so much better in myself, I intend to continue treatment as long [sic] as can.

## Data Availability

The original contributions presented in the study are included in the article/Supplementary Material, further inquiries can be directed to the corresponding author.
